# Data set of intrinsically disordered proteins analysed at a local protein conformation level

**DOI:** 10.1016/j.dib.2020.105383

**Published:** 2020-03-05

**Authors:** Akhila Melarkode Vattekatte, Tarun Jairaj Narwani, Aline Floch, Mirjana Maljković, Soubika Bisoo, Nicolas K. Shinada, Agata Kranjc, Jean-Christophe Gelly, Narayanaswamy Srinivasan, Nenad Mitić, Alexandre G. de Brevern

**Affiliations:** a Biologie Intégrée du Globule Rouge UMR_S1134, Inserm, Univ. Paris, Univ. de la Réunion, Univ. des Antilles, F-75739 Paris, France; b Laboratoire d'Excellence GR-Ex, F-75739 Paris, France; c Faculté des Sciences et Technologies, Saint Denis Messag, F-97715 La Réunion, France; d Institut National de la Transfusion Sanguine (INTS), F-75739 Paris, France; e Etablissement Français du Sang Ile de France, Créteil, France; f IMRB - INSERM U955 Team 2, Transfusion et maladies du globule rouge, Paris Est- Créteil Univ., Créteil, France; g UPEC, Université Paris Est-Créteil, Créteil, France; h University of Belgrade, Faculty of Mathematics, Belgrade, Serbia; i Discngine, SAS, 75012 Paris, France; j SBX Corp., Tōkyō-to, Shinagawa-ku, Tōkyō, Japan; k IBL, F-75015 Paris, France; l Molecular Biophysics Unit, IISc, Bangalore, India

**Keywords:** Protein disorder, PDB, Ensembles, Entropy, Local protein conformation, Structural alphabet

## Abstract

Intrinsic Disorder Proteins (IDPs) have become a hot topic since their characterisation in the 90s. The data presented in this article are related to our research entitled “A structural entropy index to analyse local conformations in Intrinsically Disordered Proteins” published in Journal of Structural Biology [1]. In this study, we quantified, for the first time, continuum from rigidity to flexibility and finally disorder. Non-disordered regions were also highlighted in the ensemble of disordered proteins. This work was done using the Protein Ensemble Database (PED), which is a useful database collecting series of protein structures considered as IDPs. The data set consists of a collection of cleaned protein files in classical pdb format that can be readily used as an input with most automatic analysis software. The accompanying data include the coding of all structural information in terms of a structural alphabet, namely Protein Blocks (PBs). An entropy index derived from PBs that allows apprehending the continuum between protein rigidity to flexibility to disorder is included, with information from secondary structure assignment, protein accessibility and prediction of disorder from the sequences. The data may be used for further structural bioinformatics studies of IDPs. It can also be used as a benchmark for evaluating disorder prediction methods.

**Table:** **Specification Table**

Subject	Biochemistry.
Specific subject area	Structural Bioinformatics, proteins disorder.
Type of data	A collection of atom coordinates in the pdb format, tables, text files and Figures.
How data were acquired	A survey of the Protein Ensemble Database (PED).
Data format	Raw, analysed and filtered
Parameters for data collection	A Protein Ensemble Database survey was performed in march 2019. The data set consists of PED stores 25,473 protein structures of 60 ensembles in 24 entries in the Protein Data Bank (pdb) format. The atom coordinate files were cleaned and treated as described below and as such may be used for further automatic analysis.
Description of data collection	Every entry of PED was analysed, i.e. some have inconsistencies. Then, all cleaned files were used for Protein Blocks (PBs) assignment, the frequency of each PBs was calculated, and local entropy was computed. All files are provided. In a similar way, DSSP was used to assign secondary structure and solvent accessibility for each residue. The dataset also collects disorder prediction generated from DisoPred and PrDOS webserver. Flat text files are provided for simple use and some Figures for better visualisation.
Data source location	University of Paris, Paris, France.
Data accessibility	Data is given in the paper. It can as well be downloaded from: http://www.dsimb.inserm.fr/∼debrevern/RESEARCH/IDP-PB/.
Related research article	Akhila Melarkode Vattekatte, Tarun Jairaj Narwani, Aline Floch, Mirjana Maljković, Soubika Bisoo, Nicolas K. Shinada, Agata Kranjc, Jean-Christophe Gelly, Narayanaswamy Srinivasan, Nenad Mitić & Alexandre G. de Brevern, (2020) “A structural entropy index to analyse local conformations in Intrinsically Disordered Proteins”, *Journal of Structural Biology*, in press [1].

**Value of the Data**

• Atomic coordinate files in pdb format are processed in a manner suitable for most analysis programs.
• The PB assignment and entropy calculation allow defining the rigidity – flexibility – disordered state as done in [1] and are easy to use for further research.
• The secondary structure assignment and solvent accessibility are provided, as they represent the basis for structural analyses.
• Two types of disorder prediction methodologies are provided; all these data can be used as a benchmark for evaluating disorder prediction methods.
• These data were largely used for the *Journal of Structural Biology* [1], and can be useful for researchers interested in the analyses of IDPs and IDRs, but also for the development of novel prediction approaches. The addition of secondary structure assignment, solvent accessibility and the two different disorder prediction methodologies will also help them greatly.

## 1 Data description

Intrinsic Disorder Proteins (IDPs) and Intrinsic Disorder Regions (IDRs) are a non-negligible part of the protein structures. IDPs are not ordered and are likely to be unfolded in solution under native functional conditions [2], [3], [4]. They do not have a well-defined 3-D structure, but embrace an ensemble of conformations. In our recent research [1], we have analysed the Protein Ensemble Database (PED3) [5] in the light of a structural alphabet [6]. PED3 is a useful database collecting series of protein structures associated to IDPs. PED stores 25,473 protein structures of 60 ensembles in 24 entries.

We provide the entire dataset in four separate folders. The data collected in these folders represent the core of our previous research published in the *Journal of Structural Biology* [1].

The first folder (1_DATA) consists of the raw data, i.e. the 24 entries with accompanying ensembles in the pdb format. They could be directly downloaded from PED website, but we have cleaned few of them for better parsing. Each subdirectories is noted PED*x*AA*y*-pdb, where *x* is always a number ranging from 1 to 9, and *y* is a letter ranging from A to D, i.e. PED1AAD-pdb (β-synuclein).

The second folder (2_PBs) corresponds to the local protein conformations analyses in the light of Protein Blocks (PBs, [7]). PBxplore software [8] was used to translate the protein structures in terms of PBs. For each entry, text files are provided with corresponding Figures. The name of directories follows the same rules with PED*x*AA*y*. For each structure entry, the pdb files assigned as series of PBs are named PED*x*AA*y*.PB.fasta. When multiple chains are found, the syntax is slightly changed to PED*x*AA*y*-chain*Z*.PB.fasta, with chain *Z* added in the name. PBs are small prototypes of 5 residues length, ranging from *a* to *p*. The first two and the last two residues are not assigned and are labelled *Z*. In rare cases, too many residues are incomplete in the pdb file, therefore only stretches of *Z* are assigned by the PBxplore.

From the distribution of PBs, the frequencies of every PB at a given position are computed and saved in PED*x*AA*y*.PB.count files. These frequencies are used to compute an entropy index named *N*_eq_ which defines whether the position is rigid, flexible or disordered. The entropy index is stored in the files named PED*x*AA*y*.PB.Neq. This information is easily readable and parsable for future analyses; visual representations are given with corresponding Figures. Firstly, one PB frequency map is shown (files named PED*x*AA*y*.map.png). In this map the colours range from deep blue (lack of a given type of PB) to red (only one type of PB) for a fixed residue position, with a grading of green, yellow and orange for intermediate states. Secondly, the same information is also shown with logos of PBs, the logo sizes are proportionate to their frequencies (files named PED*x*AA*y*.PB.logo.png). Finally, different 3D visualisations done with PyMOL software [9] are provided with three different protein orientations (files named PyMOL_PED*x*AA*y*.png).

The third folder (3_DDSP) corresponds to the secondary structure assignment performed with DSSP software [10]. DSSP provides the 8-states assignment (α-helix, π-helix, 3.10 helix, bend, turn, β-bridge, β-sheet and coil), but also the solvent accessibility. These two pieces of information are essential for most structural analyses. Each structure is in a file named PED*x*AA*y*-*n*.dssp, with *n* corresponding to the number of the models. DSSP is the most widely used secondary structure assignment for over thirty years.

The fourth folder (4_DISORDER) contains the disorder prediction outputs. Two very different methodologies were chosen, namely DisoPred 3.1 [11] and PrDOS [12]. Their results can be quite dissimilar. It underlines the importance to have a better description of the disorder states. Each of the 24 entries is shown individually. DisoPred subdirectory contains files named name.pbat that include prediction values of protein binding residues in disordered regions as well as disordered and ordered residues). In addition, it includes in a corresponding csv file (name.csv) and a simplified version (files named name.comb). An illustrative Figure named annotationGrid.png shows the results of DisoPred analysis. In PrDOS subdirectory a csv file summarizes all the results (prdos.name.csv), a separate plot shows the predicted values along the sequence (in png format). The whole output of the PrDOS, providing the information on the analysed protein sequence, turn available also on the website (files named *x*AA*y*-PrDOS.jpeg).

These data were therefore used for the work presented in *Journal of Structural Biology* paper [1]. They are presented in a way that can be easily reused by researchers. Adding to the PB analyses, the data of secondary structures, of accessibility to the solvent and of the prediction methods is useful in the context of the development of new methodologies for predicting disorder and/or protein flexibility.

## 2 Experimental design, materials, and methods

### 2.1 Raw data

The raw data were downloaded from PED website and correspond to an important occurrence of ensembles. PED^3^ contains 25,473 protein structures of 60 ensembles in 24 entries. Out of these, 6 entries have data from both SAXS and NMR, 7 from only SAXS, 10 from only NMR and one from Molecular Dynamics. Some entries have 10 or fewer models, while 8 have them more than 500. The PED4AAB entry, the Sendai virus phosphoprotein ensemble is the most populated with 13,718 models. All the models follow the classical PDB format (without most of the remarks). It can already be seen that some residues are incomplete and could be problematic for the future analyses.

### 2.2 Protein blocks

Protein Blocks (PBs) is a structural alphabet composed of 16 local prototypes [7], PBs are employed to analyse local conformations. Each specific PB is characterized by the φ, ψ dihedral angles of five consecutive residues. The PBs *m* and *d* can be roughly described as prototypes for central α-helix and central β-strand, respectively. PBs *a* through *c* primarily represent the N-cap region of β-strand while PBs *e* and *f* correspond to the C-caps; PBs *g* through *j* are specific to coils, *k* and *l* correspond to the N-cap region of α-helix, and PBs *n* through p to that of C-caps [6,13]. PB assignment was carried out for every residue from every snapshot extracted from MD simulations using PBxplore tool [8] available at GitHub (https://github.com/pierrepo/PBxplore). A useful measure to quantify the flexibility of each amino acid, called *N*_eq_ (for equivalent number of PBs) [7] was used. *N*_eq_ is a statistical measurement similar to entropy; it represents the average number of PBs a residue may adopt at a given position. *N_eq_* is calculated as follows [7]:

(1) $$N_{eq}=\text{exp}\left(-\sum_{x=1}^{16}f_{x}\text{ln}f_{x}\right)$$

Where, *f_x_* is the frequency of PB *x* in the position of interest. A *N_eq_* value of 1 indicates that only one type of PB is observed, while a value of 16 is equivalent to an equal probability for each of the 16 states, i.e. random distribution. We have also computed average *N_eq_* values. PBs were successfully used for the analysis of molecular dynamics simulation of e.g. integrins, Duffy Antigen Chemokine Receptor (DARC) protein, KiSS1-derived peptide receptor (KISS1R), HIV-1 capsid protein, α-1,4-glycosidic hydrolase, NMDA Receptor Channel Gate.

### 2.3 Secondary structure assignment

Secondary structure assignment was performed using DSSP [10] (DSSP 2015 version 2.2.1; the latest DSSP distribution is available at GitHub on address https://github.com/cmbi/xssp) with default parameters [14]. DSSP assigns 8-secondary structure states, with 3 helical states, namely α-, 3_10_- and π-helices, 2 definition of β-turns, namely turns (with hydrogen bonds) and bends (without hydrogen bonds), the rare β-bridge, and the frequent β-strand composing the β-sheet, and the coil (or loop) state.

### 2.4 Disorder prediction

Two approaches were used, namely DisoPred 3.1 [11] and PrDOS [12]. The first is one of the most well-known and used approaches (664 citations in January-2020 as measured by Google Scholar), the second one is less well-known but also has a large number of citations (463 at the same period). Both are based on very different approaches and provide slightly different tendencies depending on the entries, making them useful to enrich the analyses.

## Appendix Supplementary materials

Image, application 1
